# Impact of tidal fluctuations on bacterial community structure in Wuyuan Bay: A comparative analysis of waters inside and outside the tidal barrage

**DOI:** 10.1371/journal.pone.0312283

**Published:** 2024-10-25

**Authors:** Dandan Xie, Chen Feng, Jiehua Hu, Huina Lin, Hong Luo, Qi Zhang, Haibin He

**Affiliations:** 1 Collaborative Innovation Center for Intelligent Fishery, Higher Vocational College of Fujian Province, Xiamen Ocean Vocational College, Xiamen, China; 2 Xiamen Cloud Whale Ecological Environment Co., LTD, Xiamen, China; 3 College of Tea and Food, Wuyi University, Wuyishan, China; 4 Key Laboratory of Agroecological Processing and Safety Monitoring of Fujian Province, Fujian Agriculture and Forestry University, Fuzhou, China; University of Peshawar National Centre of Excellence in Geology, PAKISTAN

## Abstract

The tidal barrage at Wuyuan Bay effectively mitigated the odor from the tidal flat during ebb tide, however, its effect on bacterial community structure in waters are still unclear. In this study, high-throughput sequencing was used to analyze the structure of the microbial community in waters inside and outside the tidal barrage during flood and ebb tides. Results showed bacterial diversity was higher in water outside the barrage during flood tide. The dominated species at phylum and genus levels were various in waters inside and outside the tidal barrage during flood and ebb tides. The water inside during ebb tide (E1) were dominated by two cyanobacterial genera, *Cyanobium_PCC-6307* (42.90%) and *Synechococcus_CC9902* (12.56%). The microbial function, such as porphyrin and chlorophyll metabolism and photosynthesis, were increased in E1. *Norank_f__Nitriliruptoraceae* was identified as differential microorganism in waters inside the barrage. Inorganic nitrogen and nonionic ammonia were significantly high in E1, and were negatively correlated with *norank_f__Nitriliruptoraceae*. These results suggest tidal barrage blocks water exchange, resulting in the accumulation of nutrients in Wuyuan Bay. Consequently, the environment became favorable for the growth of cyanobacteria, leading to the dominance of algae in the water inside the barrage and posing the risk of cyanobacterial bloom. Higher Nitriliruptoraceae inside the barrage might be a cue for the change of water quality.

## Introduction

**Fig 1:**
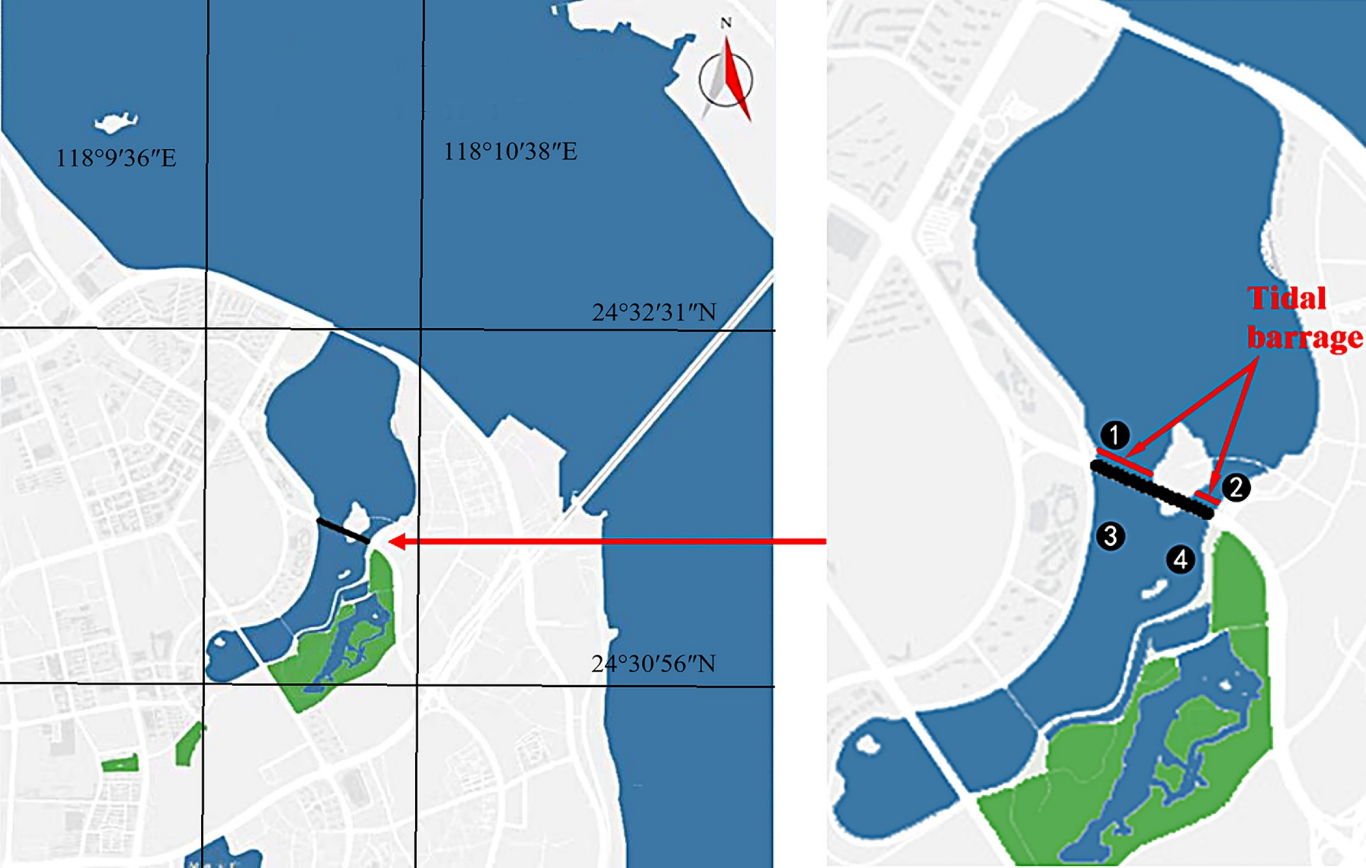
Schematic diagram of the location of the tidal barrage in Wuyuan Bay (modified from Earth Resources Observatory and Science Center, public domain). The 1, 2 are sample points of outside the barrage, and 3, 4 are sample points of inside the barrage.

Wuyuan Bay, formerly known as Zhongzhai Bay, is a natural bay in the northeast of Xiamen Island. The Wuyuan Bay area has historically been dominated by industries such as agriculture, salt production, and aquaculture; the latter have contributed to coastal erosion and seawater pollution that restricted the development of the Xiamen Special Economic Zone. In 2002, the Xiamen Municipal Government initiated a comprehensive endeavor encompassing ecological restoration, shoreline renovation, and overall development of Wuyuan Bay, resulting in a substantial enhancement of environmental quality. However, historical factors led to an incomplete underground sewerage network around the bay, causing the mixing and discharge of rainwater, domestic sewage, and industrial wastewater into Wuyuan Bay, resulting in pollutant deposition. During ebb tides, the exposed tidal flat of the bay emitted an unpleasant odor under sunlight. In May 2016, the municipal government launched the Wuyuan Bay Watershed Improvement Project that involved constructing a three-meter-high tidal barrage near the Tianyuan Bridge at the junction of the inner and outer bay (Fig 1). This barrage allowed seawater to flow into the inner bay during flood tides while blocking seawater during ebb tides to raise the water level in the inner bay to three meters. This increased the water surface area and volume to ensure continuous coverage of the tidal flat with seawater. Two three-hole sluices were built on the east and west sides of the tidal barrage to regulate the water level and ensure the safety of the tidal barrage. The holes were 7 m in width, and the total width of the six holes was 42 m. The project was completed in April 2017 and has been in operation for more than six years. Public concern about the environmental conditions of Wuyuan Bay has prompted ongoing research, primarily focused on physicochemical and ecological indicators [1, 2]. Our previously research showed that the floating debris, dissolved oxygen, water temperature, and pH in Wuyuan Bay met the Class III seawater quality standards outlined in Xiamen’s environmental function planning. However, the water showed abnormal color and odor, elevated levels of nitrogen salts, phosphorus salts, and organic matter, and a eutrophication index of 135, indicating severe eutrophication [2].

Chemical elements such as nitrogen, phosphorous, and carbon (organic matter) are the predominant nutrient for organism in nature. However, excessive nutrients in water can cause water eutrophication, accompanied by water quality deterioration [3–9]. Nutriment change in seawater could the change of microbial community structure, as well as their functions. More, many environmental factors, such as pH value, temperature, dissolve oxygen, turbidity, salinity, tidal cycles, and spatio-temporal variation etc. also played important roles in influencing microbial community structure and functional properties [10–14]. As water level fluctuations of the Poyang Lake, ɑ-diversity, functional redundancy, taxonomic dissimilarities, and taxa niche width were higher in dry-season, while functional dissimilarities were higher in wet-season [15]. The bacterial communities within the lake are highly susceptible to nutrient states and low dissolve oxygen during the rainy season [12]. Bacterial communities were taxonomically sensitive in the inundated areas and functionally sensitive in the emerged areas [11]. Ruiz-Gonzalez et al. [16] reported that large river dam regulated bacterioplankton community structure, and much greater number of bacteria particles colonized in upstream waters compared to downstream waters. Crevecoeur et al. [14] found that the aquatic microbiome was structured along the flow path and influenced mainly by higher nutrient concentrations (upstream in the Thames River) and higher temperature and pH (downstream in Lake St. Clair and Lake Erie).

It has been six years since the establishment of the tidal barrage, and the waters inside and outside the barrage undergo daily tidal cycles. Despite the daily water exchange, the water inside the barrage has a relatively stable local aquatic environment with significantly different water quality compared to the water outside the barrage [2]. However, the structure of the microbial community in the water inside and outside the barrage along with the correlations with water physicochemical indicators have remained uncharacterized. Therefore, this study utilized high-throughput sequencing to analyze and compare the bacterial diversity and community structure inside and outside the tidal barrage in Wuyuan Bay in response to tidal cycles. Additionally, we identified differential microorganisms and analyzed their correlations with water quality indicators. This study may likely helpful to water quality control and comprehensive management of the water environment in Wuyuan Bay. In a more general sense, understanding the biological and physicochemical consequences of introducing physical structures into a limited watershed may enlighten us conceiving more prudent attitude about watershed improvement project.

## Materials and methods

### Sample collection and processing

Water samples were collected inside and outside the barrage during flood tides and ebb tides (Fig 1). Specifically, water samples collected from inside and outside the barrage during flood tides were labeled as F1 and F2, respectively. Water samples collected from inside and outside the barrage during ebb tides were labeled as E1 and E2, respectively. Three replicate samples were collected. On a clean bench, each 500 mL sample was passed through a 0.22 μm filter membrane. The membrane was placed in a sterile centrifuge tube and stored in a refrigerator at −80°C for DNA extraction and high-throughput sequencing.

### Physicochemical indicators of water quality

Water quality analysis was performed according to the methods in the *Specification for Marine Monitoring* (GB17378-2007) [17], *Specifications for Oceanographic Survey* (GB12763-2007) [18], and *Sea Water Quality Standard* (GB3097-1997) [19].

### DNA extraction, high-throughput sequencing, and bioinformatic analysis

DNA extraction, high-throughput sequencing, and bioinformatic analyses were conducted by Majorbio Biopharm Technology Co., Ltd. (Shanghai, China). The procedure is briefly described below.

Total microbial genomic DNA was extracted using the E.Z.N.A.® soil DNA Kit (Omega Bio-tek, Norcross, GA, U.S.) according to manufacturer’s instructions. DNA purity was determined using a Nanodrop spectrophotometer (Thermo Scientific, United States). The DNA concentration was diluted to 100 ng μL^−1^, and DNA quality was detected by 1% agarose gel electrophoresis. The DNA samples were stored at −80°C for later use.

PCR amplification was performed using the following primers with barcodes: 341F (CCTACGGGGNGGCWGCAG) and 806R (GGACTACHVGGGGTATCTAAT). PCR was performed using TransGen AP221-02: TransStart^®^ FastPfu DNA Polymerase (ABI GeneAmp^®^ 9700). Three replicates were used for each sample. PCR products of the same sample were combined and detected by 2% agarose gel electrophoresis. The PCR products were recovered using an AxyPrepDNA Gel Extraction Kit (AXYGEN), eluted with Tris-HCl buffer, and detected by 2% agarose gel electrophoresis. The PCR products were quantified with a QuantiFluor™-ST blue fluorescence quantitative system (Promega). Libraries were constructed by Illumina bridge PCR and quantified by fluorescence using Qubit 3.0. The qualified libraries were subjected to high-throughput sequencing on an Illumina MiSeq PE300 platform.

The paired-end reads from the Illumina sequencing were merged based on overlap. Quality control and filtering of sequences were performed using Pear (v0.9.6) software. Non-repetitive sequences were clustered at 97% similarity into operational taxonomic units (OTUs) use Uparse algorithm of Vsearch (v2.7.1) software [20]. The BLAST tool was used to classify all sequences into different taxonomic groups against GenBank non-redundant nucleotide (nt) database.

QIIME (v1.8.0) was used to generate rarefaction curves and to calculate the richness and diversity indices based on the OUT information. To compare the membership and structure of communities in different samples, heatmaps were generated with the top 20 OTUs using Mothur [21]. Based on the results of taxonomic annotation and relative abundance, R (v3.6.0) software was used for bar-plot diagram analysis. To examine the similarity between different samples, clustering analyses and PCA were analysed by R (v3.6.0) based on the OTU information from each sample [22]. The evolution distances between microbial communities from each sample were calculated using the the Bray Curtis algorithms and represented as an Unweighted Pair Group Method with Arithmetic Mean (UPGMA) clustering tree describing the dissimilarity (1-similarity) between multiple samples [23]. A Newick-formatted tree file was generated through this analysis.

### Data processing

SPSS 19.0 was used for the analysis of variance followed by Least Significant Difference (LSD) multiple comparison tests. The comparison of differences in microbial community structure was performed using analysis of similarities, with *p* < 0.05 indicating significant differences. The results were plotted using Origin 8.0 and Excel 2016. Rstudio 3.3 software (Boston, MA, USA) were used for principal coordinate analysis (PCoA), heat map, random forest method and correlation network heat map.

## Results

### Water quality

**Fig 2:**
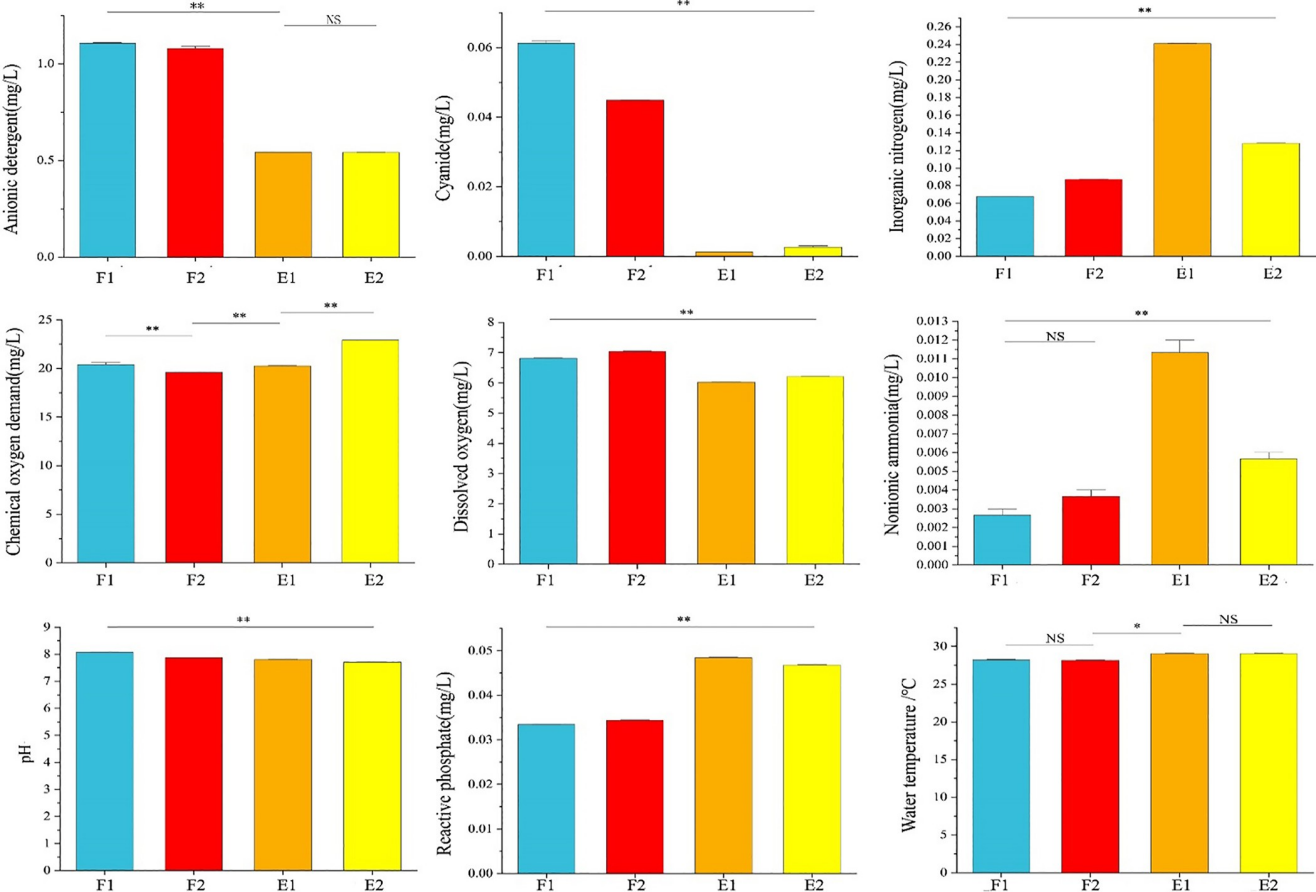
Quality indices of water samples. F1 and F2 represent samples from inside and outside the barrage at flood tide, respectively. E1 and E2 represent samples from inside and outside the barrage at ebb tide, respectively. The bars represent standard errors (± S.E.). * *p* < 0.05, ** *p* < 0.01. NS, not significantly different.

Most samples showed significant differences in water quality indicators (Fig 2). The physicochemical indicators were similar for water samples inside and outside the barrage during flood tide (F1 vs. F2). Moreover, the majority of indicators, except inorganic nitrogen, chemical oxygen demand (COD), and nonionic ammonia, demonstrated small differences between water samples inside and outside the barrage during ebb tide (E1 vs. E2). In contrast, the physicochemical indicators showed large differences between flood and ebb tides. Anionic detergent, cyanide, and dissolved oxygen were much higher during flood tide, while organic nitrogen, nonionic ammonia, and reactive phosphate were much higher during ebb tide. E1 had the highest contents of inorganic nitrogen, nonionic ammonia, and reactive phosphate, with inorganic nitrogen and nonionic ammonia nearly 4-fold those of F1, and reactive phosphate was approximately 50% higher than that of F1. E1 showed large differences in water quality compared to E2, F1, and F2.

### Analysis of sequencing data

The amplicon data of four samples comprised a total of 323,150 clean reads (133,051, 286 bp, average length of 411 bp). The sequence numbers of the four samples were in the order F2 (100,010) > F1 (79,071) > E1 (73,122) > E2 (70,947). The coverage of the four samples was all above 0.99. These results indicated adequate sequencing depth, high coverage, and reliable results. In the rarefaction curves constructed using various microbial diversity indices, the curves of all four samples leveled off, indicating that the sequencing depth was sufficient to cover the majority of microbial information in the samples. Taxonomic annotation of 1,714 OTUs revealed 34 phyla, 76 classes, 198 orders, 304 families, 492 genera, and 772 species.

### Bacterial community diversity

**Fig 3:**
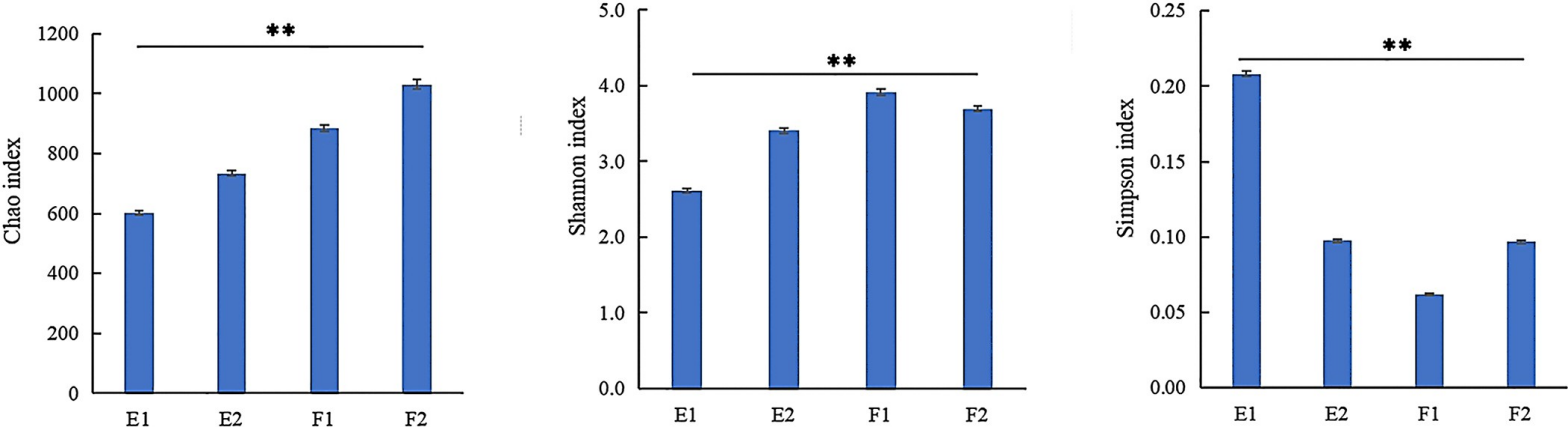
Diversity index of bacterial community in water samples. F1 and F2 represent samples from inside and outside the barrage at flood tide, respectively. E1 and E2 represent samples from inside and outside the barrage at ebb tide, respectively. The bars represent standard errors (± S.E.). ** *p* < 0.01.

The results of the community diversity analysis are presented in Fig 3. The Chao1 index followed the order of F2 > F1 > E2 > E1, indicating relatively high richness during flood tide outside the barrage. The Shannon index was in the order of F1 > F2 > E2 > E1, indicating relatively high diversity inside the barrage during flood tide and outside the barrage during ebb tide. The Simpson index was in the order E1 > E2 > F2 > F1, indicating relatively high diversity during ebb tide, outside the barrage during flood tide, and inside the barrage during ebb tide. These results suggest that both the tides and the barrage affected the bacterial community diversity, with relatively high diversity during flood tides and outside the barrage.

**Fig 4:**
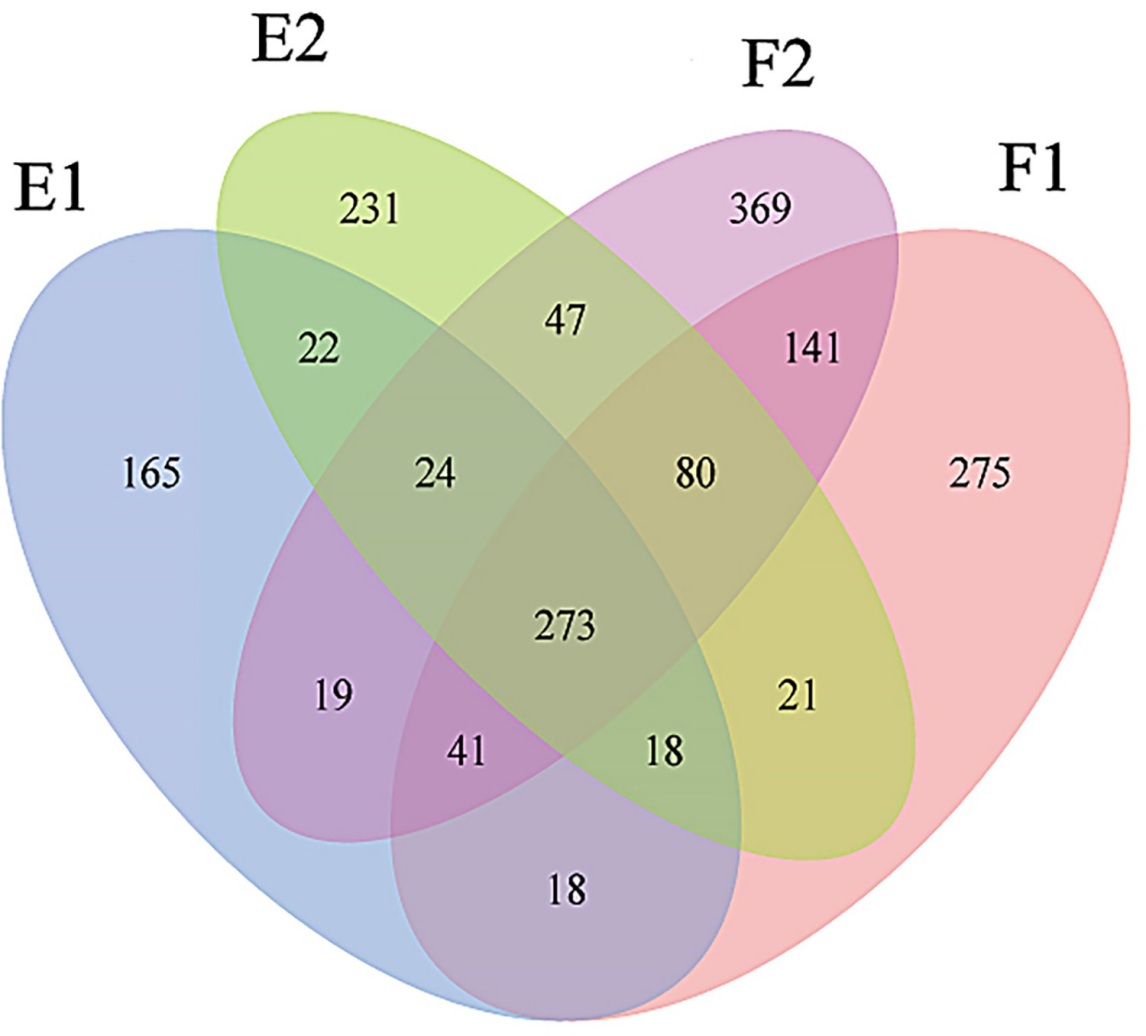
Venn analysis of OTUs. F1 and F2 represent samples from inside and outside the barrage at flood tide, respectively. E1 and E2 represent samples from inside and outside the barrage at ebb tide, respectively.

A Venn diagram (Fig 4) was prepared showing a total of 1,744 OTUs identified in the four samples. The order of F2 (994 OTUs) > F1 (867 OTUs) > E2 (716 OTUs) > E1 (580 OTUs) indicated that more OTUs were present in the samples collected during flood tides and outside the barrage. The four samples shared 273 (15.65%) OTUs. The percentages of shared OTUs were in the order E1 (47.07%) > E2 (38.13%) > F1 (31.49%) > F2 (27.46%), indicating that more shared OTUs were present in samples during the ebb tide and inside the barrage. The unique OTUs were in the order F2 (369, 37.12%) > F1 (275, 31.72%) > E2 (231, 32.26%) > E1 (165, 28.45%), indicating that more unique OTUs were present in flood tides and outside the barrage. There were 458 (26.26%) OTUs that were only present inside the barrage (E1 and F1) and 647 (37.10%) OTUs solely identified outside the barrage (E2 and F2).

Pairwise comparisons (Table 1) showed that the samples with > 60% shared OTUs (< 40% unique OTUs) were F1 (61.71%) in F1 vs. F2, E1 (61.55%) in E1 vs. F2, and E1 (60.34%) in E1 vs. F1, indicating that E1 was similar to F1 and F2. The sample with < 40% shared OTUs (> 60% unique OTUs) was F2 (shared 35.92%, unique 64.08%) in F2 vs. E1, indicating that F2 and E1 demonstrated the lowest similarity.

**Table 1:** Shared and unique OTUs in pairwise comparisons.

Shared OTUs					Unique OTUs			
F1	F2	E1	E2		F1	F2	E1	E2
/	535	350	392	**F1**	/	459/791	230/747	324/799
	53.82%	60.34%	54.75%			46.18%	39.66%	45.25%
535	/	357	424	**F2**	332/791	/	223/860	292/862
61.71%		61.55%	59.22%		38.29%		38.45%	40.78%
350	357	/	337	**E1**	517/747	637/860	/	379/622
40.37%	35.92%		47.07%		59.63%	64.08%		52.93%
392	424	337	/	**E2**	475/799	570/862	243/622	/
45.21%	42.66%	58.10%			54.79%	57.34%	41.90%	

Note: F1 and F2 represent samples from inside and outside the barrage at flood tide, respectively. E1 and E2 represent samples from inside and outside the barrage at ebb tide, respectively.

### Bacterial community structure

**Fig 5:**
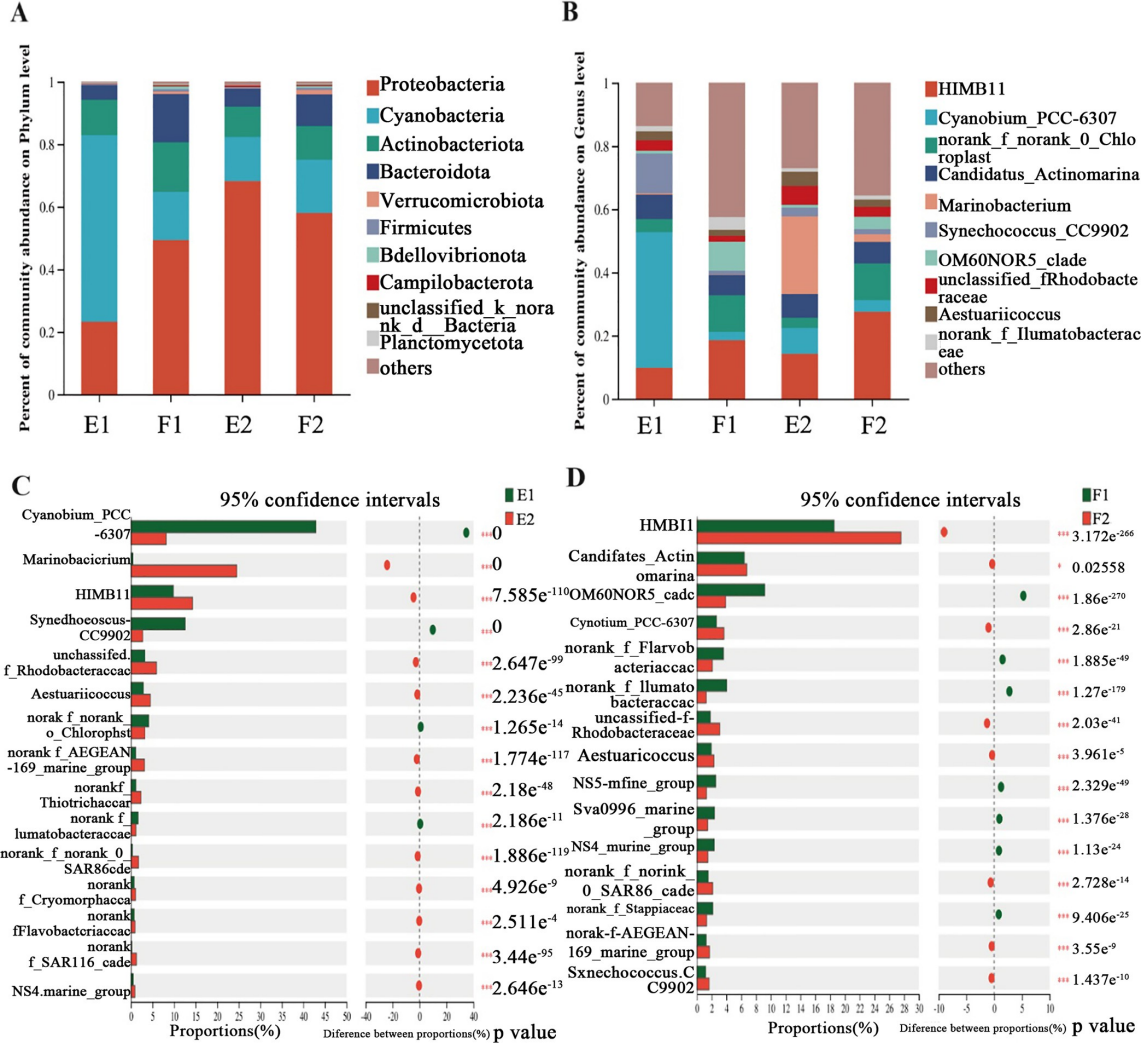
Bacterial community structures in Wuyuan Bay. The top 10 bacterial phyla (A) and genera (B) in relative abundance. Fisher’s exact test bar plot for the genus level (C and D). F1 and F2 represent samples from inside and outside the barrage at flood tide, respectively. E1 and E2 represent samples from inside and outside the barrage at ebb tide, respectively. * *p* < 0.05, *** *p* < 0.001.

At the phylum level (Fig 5A), the four samples showed similar bacterial community structure but different phylum abundance. The phyla with relatively high abundance were Proteobacteria, Cyanobacteria, Actinobacteriota, and Bacteroidota. The main phyla in F2, F1, and E2 in order were Proteobacteria > Cyanobacteria > Actinobacteriota > Bacteroidota, with Proteobacteria being the dominant phylum (58.06%, 49.32% and 68.16%, respectively). However, E1 followed the order Cyanobacteria > Proteobacteria > Actinobacteriota > Bacteroidota, with Cyanobacteria as the dominant phylum (59.64%).

At the genus level (Fig 5B), the top five bacterial genera in terms of abundance were as follows. For F1, the order was HIMB11 (18.57%) > *norank_f__norank_o__Chloroplast* (11.57%) > *OM60NOR5_clade* (9.17%) > *Candidatus_Actinomarina* (6.41%) > *norank_f__Ilumatobacteraceae* (4.03%). For F2, the order was HIMB11 (27.59%) > *norank_f__norank_o__Chloroplast* (11.62%) > *Candidatus_Actinomarina* (6.76%) > *OM60NOR5_clade* (3.90%) > *Cyanobium_PCC-6307* (3.67%). For E1, the order was *Cyanobium_PCC-6307* (42.90%) > *Synechococcus_CC9902* (12.56%) > HIMB11 (9.84%) > *Candidatus_Actinomarina* (7.78%) > *norank _f__norank_o__Chloroplast* (4.12%). For E2, the order was *Marinobacterium* (24.54%) > HIMB11 (14.29%) > *Cyanobium_PCC-6307* (8.18%) > *Candidatus_ Actinomarina* (7.52%) > *unclassified_f__Rhodobacteraceae* (5.93%). Therefore, during ebb tide, *Marinobacterium* was the dominant genus with a sharp increase in relative abundance outside the barrage, while *Cyanobium_PCC-6307* with a dramatic increase in relative abundance was the dominant genus inside the barrage. Compared with flood tides, ebb tides showed different dominant genera inside and outside the barrage, and the barrage played a critical role in determining the dominant genera inside the barrage during ebb tides.

The samples showed large differences in the relative abundance of genera (Fig 5C and 5D). Among the top five genera in terms of abundance, during ebb tide (Fig 5C), the abundances of *Cyanobium_PCC-6307* and *Synechococcus_CC9902* were significantly higher (*p* < 0.001) inside the barrage, whereas *Marinobacterium*, HIMB11 and *unclassified__f__Rhodobacteraceae* were significantly lower (*p* < 0.001) inside the barrage. During flood tide (Fig 5D), the abundances of *OM60NOR5_clade* and *norank_f__Flavobacteriaceae* were significantly higher (*p* < 0.001) inside the barrage, while those of HIMB11, *Candidatus_Actinomarina*, and *Cyanobium_PCC-6307* were significantly lower (*p* < 0.001 or *p* < 0.05) inside the barrage. These results suggest that tides significantly influenced the dominant genera, and ebb tides facilitated the growth of algae inside the barrage.

### The β-diversity of the microbial community

**Fig 6:**
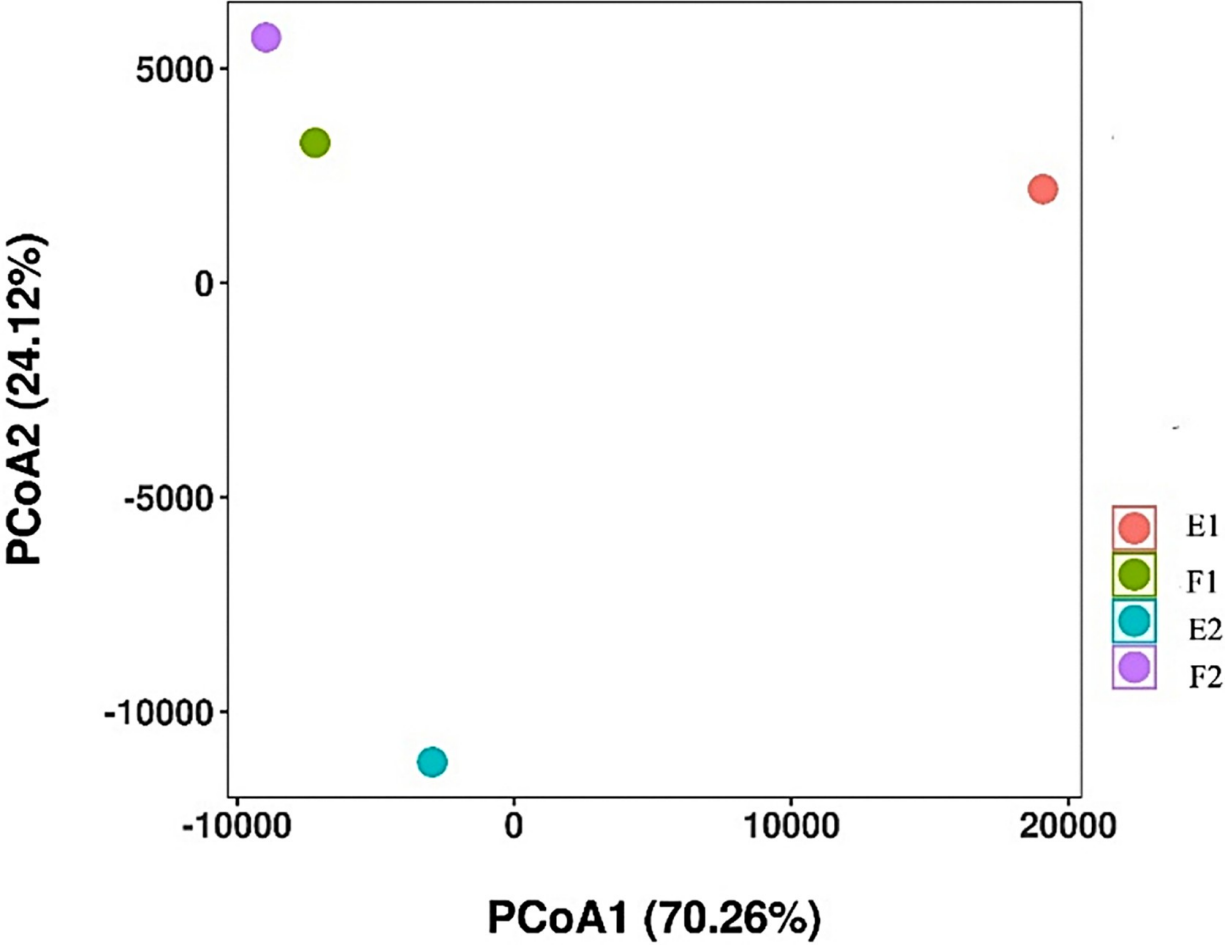
Principal coordinates analysis (PCoA) analysis of microbial communities. F1 and F2 represent samples from inside and outside the barrage at flood tide, respectively. E1 and E2 represent samples from inside and outside the barrage at ebb tide, respectively.

Principal coordinate analysis was used to distinguish the samples (Fig 6). PCoA1 and PCoA2 explained 70.26% and 24.12% of the variance, respectively. The four samples were clustered into three groups. F1 and F2 were similar at the genus level and clustered into one group, while E2 and E1 each clustered into one group. Therefore, the dominant genera inside and outside the barrage were similar during flood tides and were significantly different from those during ebb tides. The dominant genera inside and outside the barrage differed during ebb tides, and the dominant genera of E1 were the most unique.

### Microbial functions and differential microorganisms

**Fig 7:**
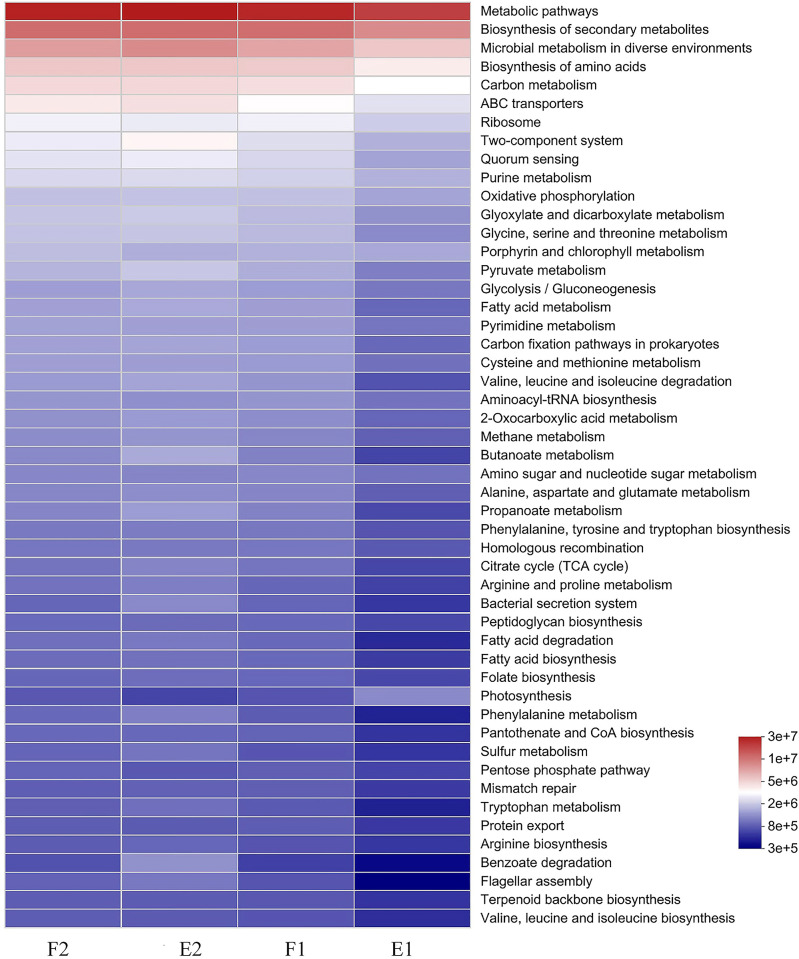
Microbial function heat map analysis. F1 and F2 represent samples from inside and outside the barrage at flood tide, respectively. E1 and E2 represent samples from inside and outside the barrage at ebb tide, respectively. Different colors represent *p*-values.

As shown in Fig 7, the top 50 functions were mainly associated with carbon and nitrogen metabolic processes, including amino acid metabolism, lipid metabolism, carbohydrate metabolism, and energy metabolism. There was little difference in microbial functions inside and outside the barrage during flood tide, with a slightly lower abundance of “ABC transporters” and “ribosome” in F1 than in F2. This may be attributed to the exchange of water inside and outside the barrage during flood tides, leading to small differences in microorganisms and microbial functions. In contrast, there were large differences in microbial functions inside and outside the barrage during ebb tides. Specifically, biosynthesis of secondary metabolites, microbial metabolism in diverse environments, biosynthesis of amino acids, carbon metabolism, ABC transporters, two-component system, quorum sensing, and bacterial secretion system as well as functions related to substance metabolism and degradation showed higher abundance in E2 than in E1. The indication is that the abundance of microbial functions related to substrate “metabolism and degradation” and “microbial metabolism in diverse environments and bacterial secretion system” decreased inside the barrage during ebb tide. Notably, the abundance of “porphyrin and chlorophyll metabolism” and “photosynthesis” was higher in E1 than in E2.

**Fig 8:**
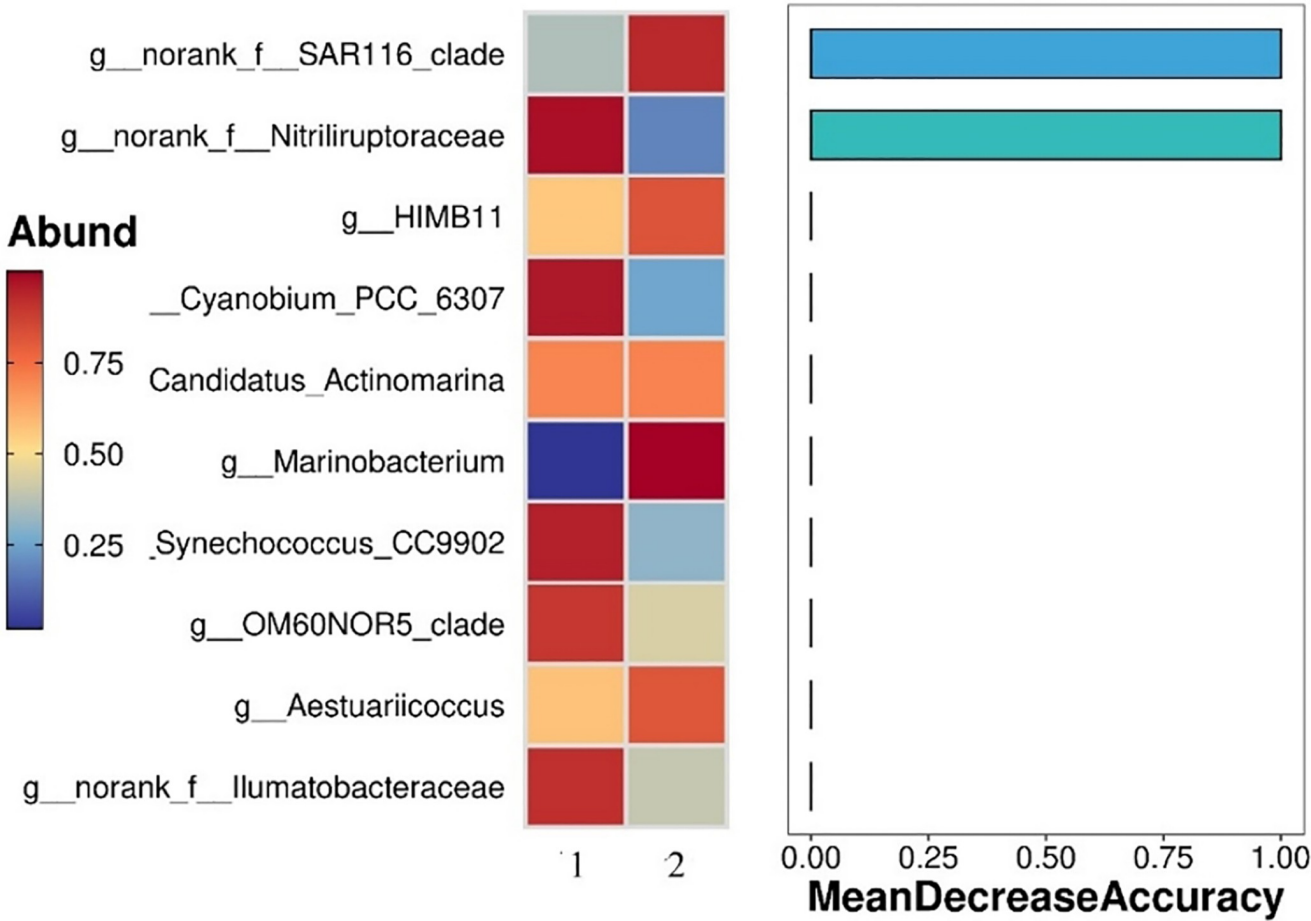
Screening differential microorganisms by random forest method. 1 and 2 represent the samples from inside and outside the barrage, respectively.

The differential microorganisms (those with a Mean Decrease Accuracy > 1) were furtherly identified by random forest analysis (Fig 8). The *norank_f__SAR116_clade* and *norank_f__Nitriliruptoraceae* had the greatest influence on the accuracy of classification between inside and outside the barrage. The abundance of *norank_f__SAR116_clade* was higher outside the barrage, while the abundance of *norank_f__Nitriliruptoraceae* was higher inside the barrage. Therefore, these two genera were identified as the differential microorganisms for outside and inside the barrage, respectively.

### Correlations between water quality indicators and differential microorganisms

**Fig 9:**
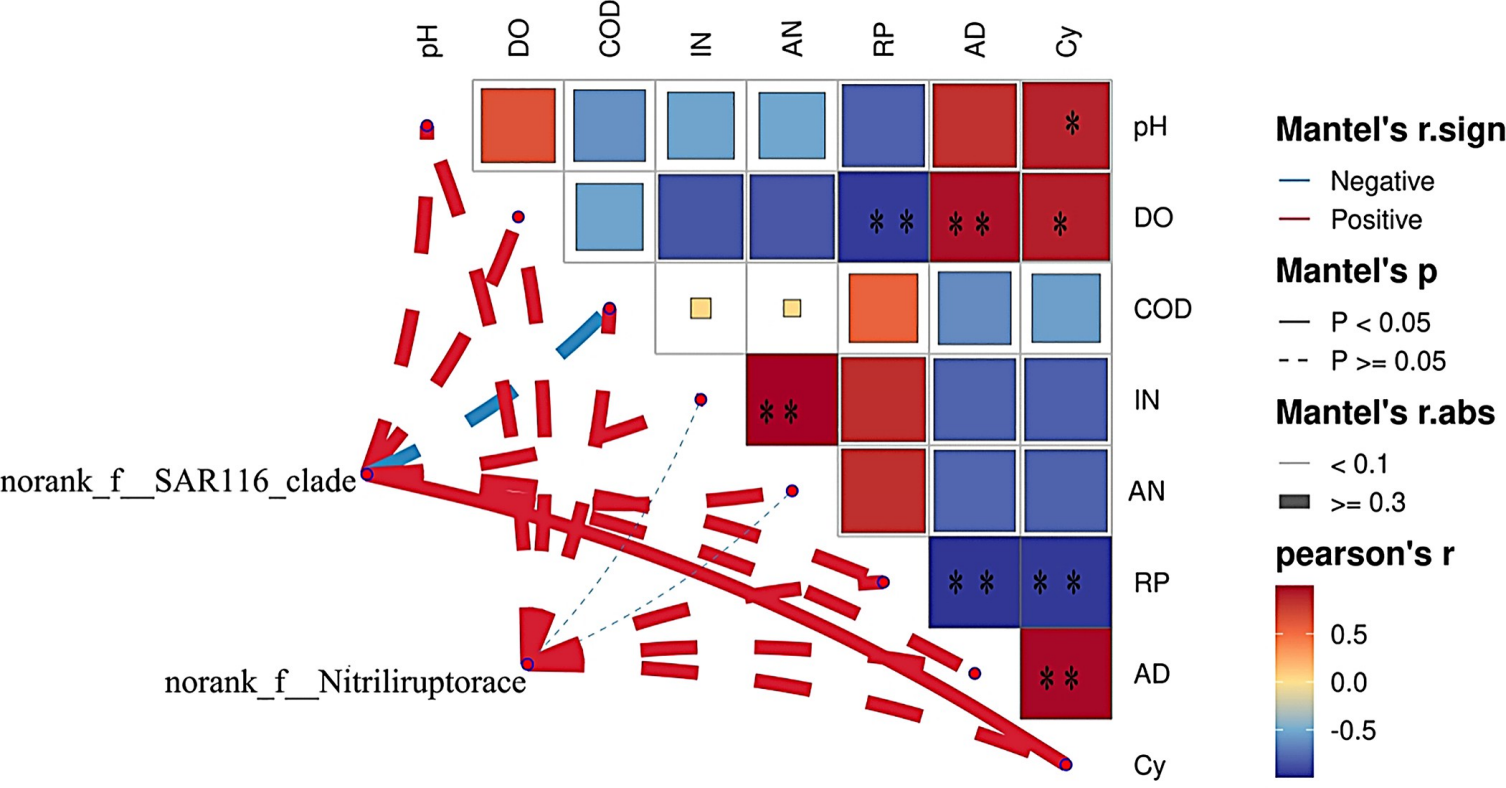
Correlation network heat map analysis of water quality index and the differential microbials. DO: dissolved oxygen, COD: chemical oxygen demand, IN: inorganic nitrogen, AN: nonionic ammonia, RP: reactive phosphate, AD: anionic detergent, Cy: cyanide. * *p* < 0.05, ** *p* < 0.01.

The results of the correlation analysis are presented in Fig 9. The water pH was significantly (*p* < 0.05) positively correlated with cyanide. Dissolved oxygen was significantly (*p* < 0.05) and highly significantly (*p* < 0.01) positively correlated with cyanide and anionic detergent, respectively, but highly significantly (*p* < 0.01) negatively correlated with reactive phosphate. Inorganic nitrogen and nonionic ammonia showed a highly significant positive correlation (*p* < 0.01). Reactive phosphate showed highly significant negative correlations with cyanide and anionic detergent (*p* < 0.01). Anionic detergent was significantly positively correlated with cyanide (*p* < 0.01).

The results of the correlation analysis between differential microorganisms and water quality indicators are shown in Fig 9. The *norank__f__SAR116_clade* was significantly (*p* < 0.05) positively correlated with cyanide and negatively correlated with COD, and positively correlated with other indicators. The *norank_f__Nitriliruptoraceae* was negatively correlated with inorganic nitrogen and nonionic ammonia but positively correlated with other indicators.

## Discussion

### Impact of the tidal barrage on water quality

The purpose of the Wuyuan Bay tidal barrage was to increase the water level in the inner bay to prevent the exposure of the tidal flat and mitigate odor emissions during ebb tide. The barrage effectively expanded the water surface area and increased the water volume during ebb tide, thereby establishing a relatively stable water ecosystem in the inner bay. This has improved the surrounding beach surface, revetment, and embankment environments, transforming the area into a new scenic park in Xiamen [1]. Nevertheless, the tidal barrage restricted the natural water flow, reduced water exchange, and caused water pollution in the inner bay [2]. Therefore, water quality and the microbial community inside and outside the barrage have been regularly monitored and assessed. Compared to the data in 2020 [2], a water quality analysis revealed the absence of floating garbage, normal color and odor, and reduced levels of inorganic nitrogen and reactive phosphate; however, there was a substantial increase in COD (Fig 2). E1 showed the highest inorganic nitrogen, nonionic ammonia, and active phosphate. Specifically, inorganic nitrogen and nonionic ammonia were nearly four times those in F1, and active phosphate was approximately 50% higher than in F1. In general, the physicochemical indicators of water samples inside and outside the barrage were similar during flood tide, while the quality of water inside the barrage during ebb tide was different from other samples. This suggests that water exchange was effective between inside and outside of the barrage during flood tide, whereas water exchange was impeded during ebb tide. Dissolved oxygen and inorganic nitrogen would be main reasons to affect the characteristics of bacterial community structure in coastal seawater [24]. Sun et al. [7] reported that nitrate-N, nitrite-N, ammonium-N, and phosphate-phosphorous were the key factors influencing bacterial communities in the aquaculture pond environment. A significant correlation was observed between inorganic nitrogen and phosphorus, and dominant bacterial genera (p < 0.05), demonstrating the potential mechanism of regulation of nutrients in bacterial communities. Li et al. [25] reported that nutrient and salinity played an important role in shaping bacterial community composition, NH_4_^+^-N and available phosphorus were the key factors in explaining the variance of the genus level. It is conceivable that the change of physiochemical indices in water inside the barrage during ebb tide will drive the changes of bacterial communities and functions.

### Microbial community structure inside and outside the barrage

The indices revealed that bacterial community diversity was relatively high during flood tides outside the barrage (Fig 3). This observation aligns with the number of OTUs identified in the four samples (Fig 4). During flood tides, seawater flows into the bay, leading to a concurrent increase in microorganisms. The barrage impeded water exchange, especially during ebb tides, resulting in the lowest community diversity being in E1. A large river dam regulated bacterioplankton community structure, caused a pronounced change of bacteria community structure in upstream and downstream waters [16]. Li et al. [26] reported that in the Three Gorges Reservoir, the Shannon-Wiener index of the bacterioplankton community in the flood season was generally higher than that in the impoundment season, suggested the element cycling and large dam disturbances are of prime importance in driving the assemblages of riverine bacterioplankton communities. Bacterial communities were taxonomically sensitive in dry-season or in the inundated areas and functionally sensitive in wet-season or in the emerged areas [11, 15]. Lo et al. [8] reported that increased total inorganic nitrogen concentrations in fish farms to favor the diverse α- and γ-proteobacteria, and bacterial community alpha diversity was positively correlated with total inorganic nitrogen.

Pairwise comparisons showed varying numbers and percentages of shared and unique OTUs (Table 1), indicating the influence of tide and barrage on microorganisms. E1 showed a similar microbial community structure as F1, F2, and E2 (Table 1), consistent with the source and water-exchange ability of microorganisms inside the barrage as well as the natural flow and exchange of seawater. F1 in the F1 vs. F2 comparison showed the least number of unique OTUs; F2 in the E1 vs. F2 comparison showed the largest number of unique OTUs, and E1 had a relatively small number of unique OTUs compared to F1 and F2 (Table 1). This can be attributed to the highest water exchange rate during F2 and the relatively stable local water environment during E1 (Fig 1). In the inner bay, the percentages of unique OTUs were 28.45% and 31.72% during ebb and flood tides, respectively (Fig 4). E1, the water sample of particular interest in this study, shared 60.34%, 61.55%, and 58.10% of OTUs with F1, F2, and E2, respectively. Moreover, E1 shared approximately 60% OTUs (i.e., 40% unique OTUs) with the other three samples (Table 1). Therefore, during ebb tide, the microbial diversity inside the barrage was primarily affected by the tide, with a relatively minor impact attributed to the tidal barrage.

### Dominant microorganisms and their functions inside and outside the tidal barrage

At the phylum level (Fig 5A), E1 was dominated by Cyanobacteria (59.64%), whereas F2, F1, and E2 were dominated by Proteobacteria (58.06%, 49.32%, and 68.16%, respectively). Proteobacteria is the largest bacterial phylum and is widespread in marine environments. Cyanobacteria is a phylum of photosynthetic autotrophs typically thriving in saline/alkaline, warm, and still waters with a high organic matter content. These bacteria are commonly found in low-salinity wetland soils [27]. In a study by Ming et al. [28], Proteobacteria was identified as the dominant bacterial phylum in both sediment and seawater samples collected from a contaminated estuary, but Cyanobacteria was only detected in seawater samples. Kolda et al. [29] used metabarcoding of cyanobacteria to study marine cyanobacteria as an ecological indicator to evaluate the coastal environments under anthropogenic stress. As shown in Fig 2, the water during ebb tide inside the barrage showed relatively high levels of nitrogen, phosphorus, and carbon sources (organic matter) that created a relatively stable environment suitable for algal growth and proliferation. Therefore, the dominance of Cyanobacteria inside the barrage may serve as an indicator of eutrophication.

At the genus level (Fig 5B), the top four dominant genera were consistent between F1 and F2, with variation only in the percentages, probably due to the sufficient exchange of water inside and outside of the barrage during flood tides. E1 and E2 shared three genera, accounting for 60.46% of E1 and 30% of E2, indicating an identical origin of these genera. Moreover, the combined abundance of the top five genera in E1 was 77.20%, and the abundance of the top genus *Cyanobium_PCC-6307* was 42.90%. This percentage was much higher than those of the other four genera, indicating that *Cyanobium_PCC-6307* was the absolute dominant genus. Moreover, the second-ranking microorganism was the algal genus *Synechococcus_CC9902* (12.56%). These findings indicate that algae dominated the water inside the barrage during ebb tide. In E2, the combined abundance of the top five genera was 60.46%, with *Marinobacterium* (24.54%) as the leading genus. The indication is that certain genera were unable to be effectively exchanged between the inner and outer bays during ebb tide, resulting in different dominant genera inside and outside the barrage. The results of cluster analysis showed that F1 and F2 were close at the genus level, while E1 and E2 showed considerable dissimilarity and formed distinct groups (Fig 6). This further confirms that the variation was relatively low during flood tide, whereas the barrage played a pivotal role during ebb tide, acting as a barrier to water exchange.

The genus *Cyanobium_PCC-6307* of cyanobacteria represents a phototrophic autotroph with the ability to effectively reduce NH_4_^+^-N in water through the assimilation and conversion of organic carbon to CO_2_ [3]. In a study of water samples from the Adriatic Sea, Kolda et al. [29] found that variation in *Cyanobium_PCC-6307*, *Synechococcus_CC9902*, and physicochemical parameters of seawater were closely related to seasonal variation in amplicon sequencing variants. Ming et al. [28] concluded that high concentrations of PO_4_^3−^ and NO_3_^−^ in heavily polluted estuarine environments led to the dominance of *Cyanobium_PCC-6307*, potentially contributing to cyanobacterial blooms. The dominance of the indicator genus *Cyanobium_PCC-6307* serves as evidence of cyanobacterial growth and blooms. *Cyanobium_PCC-6307* was the third most abundant genus in E2 (8.18%), the fifth most abundant genus in F2 (3.67%), and the most abundant genus in E1 (42.90%). This may be an indicator of a water environment favorable for the growth and proliferation of cyanobacteria, a finding that warrants special attention.

Huang et al. [13] reported that the cyanobacterial and bacterial community spatiotemporally vary along with main cyanobacterial bloom phases in upstream rivers of a eutrophicated water source reservoir. They suggested that the cyanobacterial blooms exhibit an ebb-and-flow pattern when dynamically responding to the changes of environmental factors (temperature, ammonium, nitrate, and total phosphorus etc.). Crevecoeur et al. [14] found that the same dominant bacterial phyla were detected along the water continuum of upstream in the Thames River and downstream in Lake St. Clair and Lake Erie, changing only in relative abundance. At finer taxonomical level, however, there was a clear shift in the cyanobacterial community, with Planktothrix dominating in the Thames River and Microcystis and Synechococcus in Lake St. Clair and Lake Erie. Liu et al. [11] reported that Cyanobacteria was dominant in both inundated and emerged areas, but the relative abundance of Cyanobacteria was much higher in the emerged areas than in the inundated areas. Lo et al. [8] reported that fallowed fish farms, due to the prevalence of Cyanobacteria, were a predicted high abundance of *nirA* and *narB* genes contributing to assimilatory nitrate reduction pathway. We suggest that nitrogen, phosphorus, and organic carbon and their ratios, as well as pH, temperature and dissolved oxygen are collaboration in promoting the bacterial taxonomic and functional compositional patterns in the water of inner bay.

During ebb tide, there was a notable reduction in the functions associated with carbon and nitrogen metabolism within the barrage (Fig 7). The insufficient water exchange resulted in decreases in microbial diversity as well as material metabolism and degradation, consistent with the previous analysis of microbial diversity and community structure. Notably, “porphyrin and chlorophyll metabolism” and “photosynthesis” were more abundant in E1 than in E2, suggesting the enhancement of microbial metabolism related to porphyrin, chlorophyll, and photosynthesis. This was in line with the fact that under nutrient-rich conditions (i.e., abundant carbon, nitrogen, and phosphorus), cyanobacteria leverage photosynthesis to grow, reproduce, and establish dominance in a relatively stable water environment characterized by suitable temperature and low dissolved oxygen (Fig 2).

### Water quality inside and outside the tidal barrage and the correlations with differential microorganisms

The differentially microorganisms (those with a Mean Decrease Accuracy > 1) between inside and outside the barrage are shown in Fig 8. The differential microorganism inside the barrage, *norank_f__Nitriliruptoraceae*, was negatively correlated with inorganic nitrogen and nonionic ammonia (Fig 9). The differential microorganism outside the barrage, *norank_f__SAR116_clade*, was significantly positively correlated with cyanide and negatively correlated with COD (Fig 9). Li et al. [25] reported that under the influence of the tides, *norank_f__Nitriliruptoraceae* was one of the dominant genera in tidal freshwater wetlands of the Yellow River Delta, and that this genus played vital roles in carbon, nitrogen, and sulfur cycling as well as organic matter decomposition in the wetland ecosystem. Nitriliruptoraceae demonstrated salt tolerance and the ability to decompose carbon and nitrogen substances [10, 27]. Studies have shown that water physicochemical properties such as pH, COD, temperature, salinity, phosphorus, and nitrogen can affect or determine microbial community structure in aquatic environments [5–7, 24, 30]. Ammonium nitrogen and available phosphorus are key factors influencing the abundance of bacterial genera [25]. In a small greenhouse for *Litopenaeus vannamei* aquaculture, total nitrogen, total phosphorus, inorganic nitrogen, and inorganic phosphorus were significantly correlated with the microbial community in the water [9]. The water samples collected during ebb tide showed relatively high levels of organic nitrogen, nonionic ammonia, reactive phosphate, and organic matter as well as higher water temperatures, and low levels of dissolved oxygen, cyanide, and anionic detergent. These environmental parameters were favorable for the growth of cyanobacteria, which emerged as the dominant bacterial group and thus may be used as an indicator of nutrient enrichment and eutrophication in the inner bay. The relationship of water quality and bacterial community composition and differential microorganism in a limited watershed can be as biological indicators of water health status [31, 32]. Nitriliruptoraceae was the dominant genera in water inside the barrage, might be a cue for the change of water quality.

In summary, this study showed that the tidal barrage effectively blocked the outflow of seawater from inside the barrage and the exchange with seawater outside the barrage during ebb tide. This led to the accumulation of various nutrients in the inner bay, creating an environment favorable for the growth and reproduction of cyanobacteria. This in turn led to the changes in the composition and function of the bacterial community. Algae dominated the water inside the barrage and thus may be used as an indicator of water eutrophication. Our findings indicated a heightened risk of algal blooms due to the excessive proliferation of algae. The rapid propagation of cyanobacteria consumes dissolved oxygen in water, causing environmental problems such as low levels of dissolved oxygen, water quality deterioration, and death of aquatic organisms. Addressing these issues necessitates special attention, real-time monitoring, and the implementation of preventive measures.

## Supporting information

S1 Table (XLS)
